# Pathogenesis and Diagnostic Significance of EBV-miR-BARTs in Nasopharyngeal Carcinoma

**DOI:** 10.1155/2022/4479905

**Published:** 2022-10-03

**Authors:** Zhen Wu, Xiaoling Zhang, Pinjing Liu, Aiyou Wei, Wei Ouyang, Shengjun Xiao

**Affiliations:** ^1^Xiangya Hospital, Central South University, Changsha, Hunan 410008, China; ^2^Department of Physiology, Faculty of Basic Medical Science, Guilin Medical University, Guilin, Guangxi 541100, China; ^3^Department of Hematology, Guigang People's Hospital, Guigang, Guangxi 537100, China; ^4^Department of Obstetrics and Gynecology, The First Affiliated Hospital of Sun Yat-sen University, Guangzhou, Guangdong 510000, China; ^5^College of Nursing, Guilin Medical University, Guilin, Guangxi 541100, China; ^6^Department of Pathology, The Second Affiliated Hospital of Guilin Medical University, Guilin, Guangxi 541100, China

## Abstract

**Objective:**

Examining the role of EBV-miR-BARTs in nasopharyngeal cancer etiology and diagnosis.

**Method:**

As the subjects of this study, nasopharyngeal cancer cell lines were chosen and then randomly assigned to one of four groups: the control group, EBV-miR-BART5-3p NC, EBV-miR-BART5-3p mimics, and EBV-miR-BART5-3p inhibitor groups. Utilizing reverse transcription polymerase chain reaction, we determined the levels of gene expression in nasopharyngeal cancer cells that had been treated with EBV-miR-BART5-3p (RT-PCR). The MTT, Transwell, and scratch tests were used to determine the degree to which cells underwent apoptosis, invasion, and migration. The Western blotting method was used in order to examine the protein expression.

**Result:**

Compared with normal nasopharyngeal cells, *P* 0.05 showed that nasopharyngeal cancer cells had greater EBV-miR-BART5-3p expressions and proliferation rates in the control, EBV-miR-BART5-3p NC, and EBV-miR-BART5-3p No statistically significant differences were seen between the mimic groups (*P* > 0.05); compared with the control group, the proliferation rate of the EBV-miR-BART5-3p inhibitor group was lower with *P* < 0.05. At a significance threshold of *P* 0.05, there was no difference in the rates of apoptosis between the control group and the EBV-miR- BART5-3p NC group. Comparing the control group to the EBV-miR-BART5-3p mimics group and the EBV-miR-BART5-3p inhibitors group revealed that the rate of apoptosis was dramatically enhanced in the EBV-miR-BART5-3p inhibitors group but significantly decreased in the control group (*P* 0.05). When comparing the control group to the EBV-miR-BART5-3p NC group, there was no statistically significant change in the total number of invasive cells (*P* > 0.05). When comparing the EBV-miR-BART5-3p mimics group to the control group, we found a statistically significant increase in the former and a decrease in the latter (*P* 0.05). The migration rates of the control group, the EBV-miR-BART5-3p NC group, and the EBV-miR-BART5-3p mimics group did not vary from one another in a way that was statistically significant (*P* > 0.05). When compared to the control group, the migration rate was considerably (P 0.05) lower in the EBV-miR-BART5-3p inhibitor group. There were no discernible changes identified (*P* > 0.05) in the levels of Bcl-2 protein expression in the control group, the EBV-miR-BART5-3p NC group, and the EBV-miR-BART5-3p mimic group in a research that compared these three groups. Protein levels of BCL-2 were significantly decreased (*P* 0.05) in the EBV-miR-BART5-3p inhibitor group, in comparison to the control group. When comparing the control and EBV- miR-BART5-3p NC groups, we found no statistically significant differences in Bax and Caspase-3 protein expression levels (*P* > 0.05). The protein expressions of Bax and Caspase-3 were statistically significantly greater in the EBV-miR-BART5-3p contrast between the inhibitor and control groups. When comparing the protein expressions of MMP-2 and MMP-9 between the control group, the EBV-miR-BART5-3p NC group, and the EBV-miR-BART5-3p mimics group, there was no statistically significant change (*P* > 0.05). Protein levels of MMP-2 and MMP-9 were inhibited by EBV-miR-BART5-3p to a greater extent (*P* 0.05) in the experimental group compared to the control group.

**Conclusion:**

The understanding that inhibiting expression of EBV-miR-BART5-3p might reduce the risk of developing nasopharyngeal cancer may help direct clinical treatment for the condition.

## 1. Introduction

Nasopharyngeal carcinoma is caused by the canceration of epithelial cells of nasopharyngeal mucosa, which is mostly found in Southeast Asia and the Guangdong and Guangxi regions in southern China. The total incidence of nasopharyngeal carcinoma in China is 3.16/100,000 and the recurrence rate of the disease is high and has a poor prognosis of patients [1, 2]. It is very dangerous to people's lives, health, and wellbeing. Recent years have seen a surge in interest in the role that hereditary variables, environmental factors, Epstein-Barr virus (EBV), and other factors play in the development and progression of nasopharyngeal cancer, among which EBV is involved in the entire development of nasopharyngeal carcinoma [3, 4], especially existing largely in the early stage of epithelial tumor tissue of nasopharyngeal carcinom, stimulating the invasion and metastasis of tumor cells [5]. At present, there is still a lack of targeted drugs and new therapeutic methods that can effectively reduce the toxicity of EBV. The main treatment methods for patients with nasopharyngeal carcinoma are radiotherapy or chemotherapy drugs [6] . However, these treatments have high toxicity side effects and low cure rate, and many patients occurred the adverse reactions such as anemia and bone marrow suppression after treatment, which further increased the physical and psychological burden on patients. Therefore, finding specific and effective targets or biomarkers may be clinically significant since it holds the key to enhancing the disease's cure rate and prognostic consequences. Single-stranded, noncoding RNA microRNAs (miRNAs) are tiny molecules that may control gene expression. The research on the regulation mechanism of miRNA on tumor cells has gradually attracted attention in recent years. Based on the context, miRNAs may either promote or inhibit tumor growth. Which affects the proliferation and differentiation of tumor cells [7, 8]. EBV is a virus that can encode miRNA in host cells [9]. EBV-miR-BART survives in host cells, blocking immune response, whose cell biological behaviors have played a key role in cancer metabolism; the EBV-encoded miR-BART contributes to the onset and progression of nasopharyngeal cancer (includes BART5-5p, BART7-3p, BART5-3p, etc.). And Zheng et al. [10] found that upregulating the expression of EBV has shown that BART5-3p may both increase the number of nasopharyngeal cancer cells and prevent them from dying off naturally. So EBV-miRNA may become an effective target for the diagnosis and treatment of nasopharyngeal carcinoma by examining the expression of EBV-BART5-3p in nasopharyngeal cancer tissue and its influence on nasopharyngeal. This research was conducted to provide a scientific and clinical basis for the diagnosis and treatment of nasopharyngeal carcinoma, which is caused by carcinoma cells.

## 2. Materials and Methods

### 2.1. Cell Culture

The Chinese Academy of Sciences generously contributed both the normal nasopharyngeal cell line NP69 and CNE-2Z, a human nasopharyngeal carcinoma cell line. Cells were regularly grown and maintained in RPMI-1640 media with 10% fetal bovine serum, 100 U/ml penicillin, and 100 U/mL streptomycin, and the medium was changed every 3 days. Every other day, at a temperature of 37 degrees Celsius, a relative humidity of 95%, and a concentration of 5% carbon dioxide. When the subcultured cells were in the log phase, they were collected for harvesting.

### 2.2. Reagents and Instruments

MMT reagent (Art. No. M2128, specification 1 g), methanol (Art. No. 34860, specification 1 L), Lipofectamine ™ 2000 transfection reagent (Art. No. 1 1668030, specification 0.3 mL), and Trizol reagent (Art. No. 1 5596018, specification 2 00 mL) were all purchased from Merck Sigma Co., USA; PBS buffer (Art. No. SNM491, specification 500 mL, Beijing Biolab Technology Co., Ltd.), rabbit monoclonal Bcl-2 primary antibody (Art. No. ab182773, specification 100*μ* L), rabbit monoclonal Bax primary antibody (Art. No. ab32124, specification 100 *μ*L), rabbit monoclonal Caspase-3 (Art. No. ab32351, specification 100 *μ*L), GAPDH rabbit monoclonal primary antibody (Art. No. ab181602, specification 100 *μ*L), rabbit monoclonal MMP-2, and MMP9 (Art. No. ab92536, ab150077, specification 500 *μ*g) were all purchased from Abcam Company, UK. A purchase was made from Shanghai Yisheng Biotechnology Co., Ltd. for an Annexin V-FITC/PI apoptosis detection kit with the item number 40302ES20; an ECL luminescence kit (Art. No. HR0338) from Beijing Biolab Technology Co., Ltd.; a QuantStudio 3 real-time fluorescence quantitative PCR instrument from Thermo Fisher; a CytoFLEX-type flow.

### 2.3. Experimental Method

#### 2.3.1. Cell Transfection and Grouping

Logarithmically growing CNE-2Z cells were split into four groups. Control (nontransfected), EBV-miR-BART5-3p negative control (NC), Cells transfected with an inhibitor of EBV microRNA-BART5-3p (EBV-miR-BART5-3p inhibitor) were compared to cells expressing a mimic of this miRNA. Transfection was accomplished as described in the manual for the Lipofectamine TM 2000 transfection reagent.

#### 2.3.2. RT-PCR Detects EBV-miR-BART5-3p Gene

Take the CNE-2Z cells transfected under 1.2.1, add Trizol reagent to lyse for 10 min, extract the total RNA in each group of cells and reverse transcribe into cDNA, and use a PCR instrument to measure content of EBV-miR-BART5-3p. The conditions were predenaturation at 95°C for 30 s, denaturation at 95°C for 10 s, annealing at 55°C for 30 s, and extension at 70°C for 10 s for a total of 30 cycles. The PCR reaction system used U6 as the internal reference, and the 2^−ΔΔCt^ method was used for calculation. EBV-miR-BART5-upstream 3p's primer was 5′-GTGGGCCGCTGTTCACCT-3′, and its downstream primer was 5′-TGGTGTCGTGGAGTCG-3′; U6's upstream primer was 5′-CTCGCTTCGGCAGCACAT-3′, and its downstream primer was 5′-TTTGCGTGTCATCCTTGCG-3′.

#### 2.3.3. MTT Test Detects Cell Proliferation

Using a 96-well plate with CNE-2Z cells transfected at a ratio of 1.2.1 : 1.0105, add 20 l of MTT solution to each well, add 200 *μ*l of DMSO solution after shading cultured for 4 hours, and then shake to dissolve and after fully dissolving, detect the absorbance at 490 nm wavelength with a microplate reader and calculate the cell proliferative skill, and the procedure was performed three times. Formula: absorbance of cells in the experimental group/absorbance of cells in the control group = cell proliferation rate.

#### 2.3.4. Detection of the Ability of Cell Apoptosis Ability by Flow Cytometry

Take the CNE-2Z cells transfected in 1.2.1, inoculate them, respectively, in 6-well plates, and wash them with precooled PBS buffer in the refrigerator. Annexin V-FITC/PI cell apoptosis detection reagent kit instructions were followed, and the apoptosis rate (in percentage) was measured using a flow cytometer. This study was repeated thrice.

#### 2.3.5. Transwell Test Detects Cell Invasion

Take the CNE-2Z cells transfected in 1.2.1, digest the cells with trypsin and centrifuge, and then wash them once with PBS. First, set the cell concentration to 5 x 105 cells/mL. Transwell chambers covered with matrix gel were used to incubate 200 L of cells for 24 hours at 37 degrees Celsius, in a CO2 incubator. A cell culture plate containing 10% FBS medium was used to cultivate the cells. Subsequently, after removing the Transwell chamber, the matrix gel and cells within were cleaned with a cotton swab and preserved in a 4% formaldehyde solution for 10 minutes. The cells at the bottom of the well were stained for 10 minutes with 0.1% crystal violet solution before being rinsed and examined under a microscope.

#### 2.3.6. Scratch Assay Was Used to Detect Cell Migration

The cells were collected to make the cells continue to grow in a monolayer adherent state until the confluence of cells reached 80% to 90%. Then, cells were scratched by keeping the pipette perpendicular to the well plate; consistent scratch width may be achieved. Then, to get rid of any leftover debris, the cells were rinsed three times with PBS before being placed in an incubator set at 37 degrees Celsius and 5% carbon dioxide. Under the microscope, the resulting photos were taken at 0 hours and 48 hours after the experiment began. The formula for calculating the migration rate as a percentage is as follows: (24 hours minus 0 hours)/0 hours multiplied by 100 percent.

#### 2.3.7. Western-Blot Was Used to Detect the Related Protein Expression in Nasopharyngeal Carcinoma Cells

The CNE-2Z cells transfected in 1.2.1 were added to the protein lysate and extracted cells of each group after the substance was tested using a BCA kit after being centrifuged at 12,000 rpm for 10 minutes at 4 degrees Celsius. kit for protein concentration. Following a 10-minute incubation in 0.1% crystal violet solution, the cells at the bottom of the well were washed and examined under a microscope. 30 *μ*g protein was boiled and denatured in each group and made the electrophoresis in 10% polyacrylamide gel to transfer to blocked with 5% skim milk powder for 1 hour at room temperature on a PVDF membrane. The diluted Bcl-2 in primary antibodies of Bax, Caspase-3, MMP-2, and MMP-9 primary antibodies (1 : 1 000) were added and grew in a 4°C incubator overnight, rinsed three times in PBS buffer, adding diluted secondary antibodies (1 : 5000), a one-hour room temperature incubation followed by three further washings, adding ECL chemiluminescent reagent for color development, and using Quantity One software to calculate the relative expression of target protein (gray value of target protein/gray value of internal reference protein) with GAPDH as the internal reference.

### 2.4. Statistical Methods

Using SPSS22.0 software for data analysis, the measurement data conforming to the normal distribution was expressed in the form of $\left(\overset{¯}{\chi }\pm s\right)$, and multiple pairwise comparisons and one-way analysis of variance (LSD-*t* test) were utilized to examine the data from the different groups. If the *P* value is less than 0.05, the results of the study may be considered reliable.

## 3. Results

**Figure 1:**
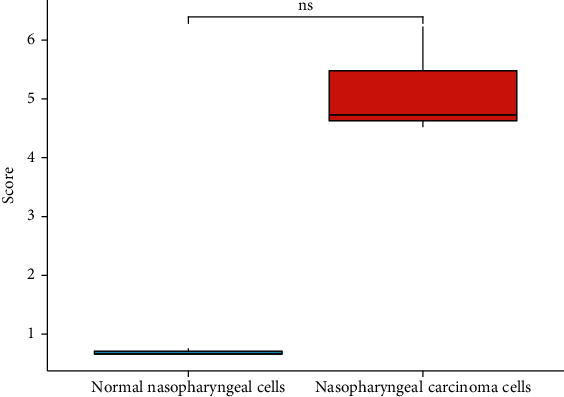
Comparison of EBV-miR-BART cells between the two groups.

Expressions of EBV-miR-BARTs in normal nasopharyngeal cells and nasopharyngeal carcinoma cells: Compared with normal nasopharyngeal cells, the Table 1 and Figure 1 reveal that EBV-miR-BART5-3p expression was significantly greater in nasopharyngeal cancer cells at *P* 0.05.

The effects of EBV-miR-BARTs on cell proliferation: There was no statistically significant difference between control, EBV-miR-BART5-3p NC, and EBV-miR-BART5-3p mimic groups in terms of proliferation rates (*P* > 0.05). The proliferation rate of the EBV-miR-BART5-3p inhibitor group was significantly lower than that of the control group (*P* 0.05; Table 2).

**Figure 2:**
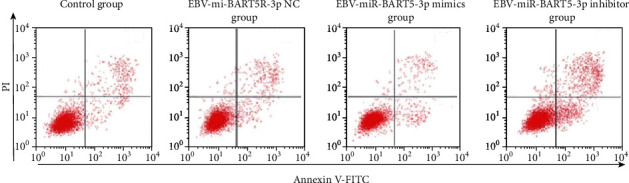
Comparison of each group's apoptosis rates.

The effects of EBV-miR-BARTs on cell apoptosis: When comparing the control group and the EBV-miR-BART5-3p NC group, there was not a statistically significant difference in apoptotic rates between the control group and the EBV-miR-BART5-3p inhibitor group (*P* > 0.05), as shown in Table 3 and Figure 2. On the other hand, the apoptosis rate of the miR-BART5-3p mimic group was lower than that of the control group.

**Figure 3:**
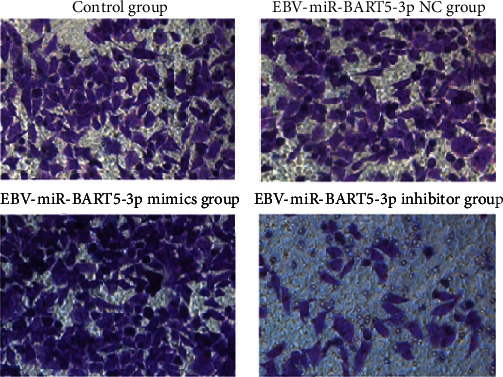
Comparison of the number of invasive cells in each group (×200).

The effects of EBV-miR-BARTs on cell invasion: Counts of invasive cells from the control and EBV-miR-BART5-3p NC. None of the groups were statistically different from one another (*P* > 0.05), but Table 4 and Figure 3 show that the number of invasive number of EBV-infected cells in the EBV-miR-BART5-3p mimics and EBV-miR-BART5-3p inhibitor groups was significantly higher than in the control group. With a 0.05 cutoff for statistical significance, the study was considered to be statistically significant.

**Figure 4:**
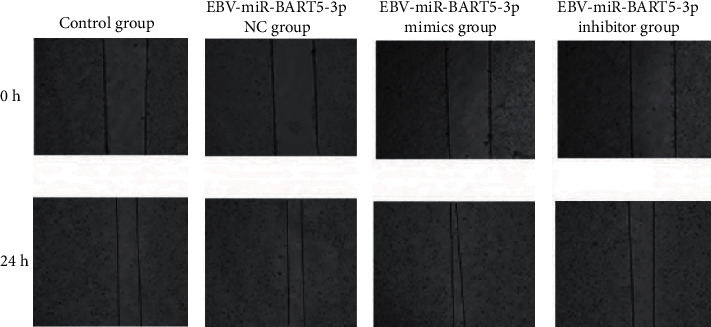
Comparison of cell migration rates in each group vs. the placebo group.

Cell migration as affected by EBV-miR-BARTs: When comparing the control group, the EBV-miR-BART5-3p NC group, and the EBV-miR-BART5-3p mimic group, there was a significant difference in the migration rates; there was no statistically significant difference (*P* > 0.05); as shown in Table 5 and Figure 4, the migration rate of the EBV-miR-BART5-3p inhibitor group was significantly lower than that of the control group (P 0.05).

**Figure 5:**
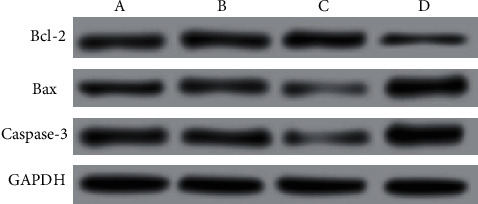
Comparison of apoptosis proteins in each group. Note: A is the control group; B is the EBV-miR-BART5-3p NC group; C is the EBV-miR-BART 5-3p mimic group; D is the EBV-miR-BART5-3p inhibitor group.

The effects of EBV-miR-BARTs on apoptosis proteins: Neither the control group nor the EBV-miR-BART5-3p NC group nor the EBV-miR-BART5-3p mimic group showed statistically significant differences in Bcl-2 expression levels (*P* > 0.05). The expression of BCL-2 protein was significantly decreased (*P* 0.05) When compared to the control group, Bax and Caspase-3 protein expressions were higher in the EBV-miR-BART5-3p inhibitor group, but there was no significant difference between the control and EBV-miR-BART5-3p NC groups (*P* > 0.05) (*P* 0.05), as shown by Table 6 and Figure 5.

**Figure 6:**
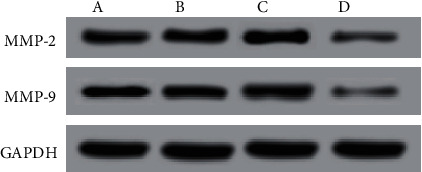
Comparison of cell invasion and apoptosis proteins in each group. Note: A is the control group; B is the EBV-miR-BART5-3p NC group; C is the EBV-miR-BART5-3p mimic group; D is the EBV-miR-BART5-3p inhibitor group.

The effects of EBV-miR-BARTs on invasion proteins: No discernable alterations in MMP-2 and MMP-9 protein expression were found between the control group, the EBV-miR-BART5-3p NC group, and the EBV-miR-BART5-3pa congregation of mimics. Protein expression levels of MMP-2 and MMP-9 were found to be considerably lower in the EBV-miR-BART5-3p inhibitor group compared to the control group (Figure 6 and Table 7).

## 4. Discussion

According to what its name says, nasopharyngeal carcinoma is a malignant epithelial tumor of the nasal and pharyngeal airways. The vast majority of cases have a pathological form that is a poorly differentiated, highly aggressive squamous cell carcinoma [11]. Because of this, it is of the utmost necessity to look into developing new treatment methods that can be used in the clinical care of patients who have this ailment. Numerous studies have shown that 44 different miR-BARTs are expressed in this disease. These miR-BARTs are responsible for oncogene or cancer suppressor gene functions, and they regulate the expressions of some EBV genes as well as host genes at the posttranscriptional level. This is an extremely important factor in the development, progression, and spread of the disease [12, 13]. Furthermore, miR-BART5-3p was shown to influence the development of the disease, but its specific mechanism is not clear, so this study took nasopharyngeal cancer cells as the research objects for analysis.

EBV-miR-BART5-3p expression was initially identified in nasopharyngeal carcinoma and normal nasopharyngeal cells. The data demonstrated that EBV-miR-BART5-3p expressions were greater in nasopharyngeal carcinoma cells compared to normal nasopharyngeal cells, suggesting that EBV-miR-BART 5-3p was really strongly expressed in the disease and was directly associated to the incidence of the illness. As we all know, the most typical and essential feature of cancer cells is proliferation, so controlling this feature can effectively prevent the occurrence of diseases. In addition, the most effective way to maintain basic cell activity is apoptosis, which is considered to be effective in eliminating cancer cells while causing very minimal damage to surrounding healthy tissue, which is also an important way for many chemotherapy drugs to clear tumor cell's way. Because it can participate in the ability of identifying the body and dealing with virus-infected cells in the body and inhibit the occurrence of cancer by regulating and improving damaged cells, so it is considered to be the key research direction of clinical treatment [14]. Therefore, this study detected the proliferative ability and apoptosis ability of nasopharyngeal carcinoma cells. According to the findings, the rates of cell proliferation in the control group, the EBV-miR-BART5-3p NC group, and the EBV-miR-BART5-3p mimic group did not significantly vary from one another. When the proliferation rate of EBV-miR-BART5-3p inhibitors was compared to that of the control group, it was shown to be lower. Flow cytometry analysis showed that comparison of apoptosis rates between the control and EBV-miR-BART5-3p NC groups revealed no statistically significant differences. While The EBV-miR-BART5-3p mimics group had considerably lower apoptosis than the control group, whereas the EBV-miR-BART5-3p inhibitors group had much more apoptosis than the control group. The fact that inhibiting its activity has the potential to effectively restrict the proliferation of nasopharyngeal carcinoma cells and promote the death of those cells demonstrates that EBV-miR-BART5-3p plays a role in the etiology of nasopharyngeal carcinoma. This is demonstrated by the fact that the inhibition of its activity has the potential to effectively restrict the proliferation of nasopharyngeal carcinoma cells. Moreover, the most harmful aspects of cancer are the disruption of the dynamic balance between cell proliferation and apoptosis, which leads to the creation and growth of malignant tumors, and life-threatening behavior of cancer cells is invasion and metastasis. It is not only a complex behavior in the process but also interfered by many factors, so it is necessary to study the mechanisms of cancer cell proliferation, invasion, and apoptosis. Therefore, this is also one of the key research directions of clinical treatment, which can inhibit the disease by controlling or blocking these steps. Therefore, this study also detected the invasion and spread of nasopharyngeal cancer cells to other parts of the body. When invasive cell counts were compared between the control and EBV-miR-BART5-3p NC groups, the findings indicated no significant difference. According to the scratch test data, the number of invasive cells was more in the EBV-miR-BART5-3p reduction in the group that was treated with the EBV-miR-BART5-3p inhibitor in comparison to the group that received control treatment. Comparing the migration rates of the BART5-3p NC group, the EBV-miR-BART5-3p NC group, and the EBV-miR-BART5-3p mimic group, there was no statistically significant difference. As expected, migrating cells were less numerous in Epstein-Barr virus-specific inhibitors of the microRNA BART5-3p. Nasopharyngeal cancer cells were shown to invade and metastasize less when this miRNA was blocked, at least in comparison to the control group.

Studies [15, 16] believe that the Bcl-2 protein family produces the apoptosis-promoting proteins and plays a crucial part in the mitochondria-mediated death process, and the apoptosis-inhibiting proteins were playing a synergistic role to regulate cell apoptosis. In addition, since the initiation of apoptosis, often known as the process of programmed cell death, it requires the presence of the enzyme Caspase-3. Regulating the synthesis of the antiapoptotic protein Bcl-2 and the activation of downstream Caspase-3 is one possible method for avoiding the death of neurons. In order to do this, apoptosis-related protein expression was evaluated. The results showed that the expression of Bcl-2 protein did not vary significantly among the Ccontrol, EBV-miR-BART5-3p NC, and EBV-miR-BART5-3p mimic groups. While compared with the control group, the BCL-2 protein expression in the EBV-miR-BART5-3p inhibitor group was lower. It was found that there was no statistically significant difference between protein expression levels of Bax and Caspase-3 in the control vs. EBV-miR-BART5-3p NC groups compared with the Control group, Bax, and Caspase-3 protein. The expressions were found to be greater in the EBV-miR-BART5-3p inhibitor group. This suggests that inhibiting the expression of EBV-miR-BART5-3p can promote the apoptosis of nasopharyngeal carcinoma cells by downregulating Bcl-2 protein and upregulating Bax and Caspase-3 protein expressions. Besides, one of the basic loss of extracellular matrix is a necessary prerequisite for tumor spread. MMP is an extracellular matrix degradation enzyme whose main members MMP-2 and MMP-9 are the most critical proteins involved in tumor cell invasion, so the increased expression of MMPs may increase the invasion and metastasis of malignancies [17–19]. Therefore, additionally, invasion-related protein expression was detected, and the findings demonstrated that comparison of MMP-2 and MMP-9 protein expressions among the control, EBV-miR-BART5-3p NC, and EBV-miR-BART5-3p mimic groups revealed no statistically significant differences. The levels of protein expression for MMP-2 and MMP-9 were lower in the group that was given the EBV-miR-BART5-3p inhibitor compared to the group that was given the control. Based on these findings, it seems that EBV-miR-BART5-3p at low concentrations may limit MMP production, preventing cell invasion and metastasis by reducing MMP-2 and MMP-9 expression.

In conclusion, upregulating the EBV-miR-BART5-3p can regulate the occurrence and development of nasopharyngeal carcinoma cells, indicating that the mechanism through which EBV-miR-BARTs has a role in illness is still unknown. By making an impact on cell biology by controlling related apoptosis and invasion proteins. Blocking EBV-miR-BART5-3p expression reduces the risk of new cases of Epstein-Barr virus infection and further deterioration of nasopharyngeal carcinoma, suggesting that EBV-miR-BARTs may become a new biomarker for targeted therapy of nasopharyngeal carcinoma, which provides a certain reference for clinical treatment. However, its pathways and targets have not been fully elucidated, and further research is needed to reveal it.

## Figures and Tables

**Table 1:** Comparison of EBV-miR-BART5-3p gene levels between nasopharyngeal carcinoma and nasopharyngeal mucosal inflammatory tissue samples $\left(\overset{¯}{\chi }\pm s\right)$.

Grouping	*n*	EBV-miR-BART5-3p
Normal nasopharyngeal cells	3	0.69 ± 0.06
Nasopharyngeal carcinoma cells	3	5.16 ± 0.93
*t*		8.280
*P*		0.014

**Table 2:** Comparison of cell proliferation rates in each group $\left(\overset{¯}{\chi }\pm s\right)$.

Grouping	*n*	Proliferation rate (%)
Control group	3	88.52 ± 12.05
EBV-miR-BART5-3p NC group	3	87.14 ± 11.53
EBV-miR-BART5-3p mimics group	3	92.51 ± 8.69
EBV-miR-BART5-3p inhibitor group	3	35.80 ± 5.69^∗^
*F*		22.482
*P*		<0.001

Note: Contrasted with the negative control group, ^∗^*P* 0.05.

**Table 3:** Comparison of apoptosis rates in each group $\left(\overset{¯}{\chi }\pm s\right)$.

Grouping	*n*	Apoptosis rate (%)
Control group	3	16.85 ± 1.05
EBV-miR-BART5-3p NC group	3	16.09 ± 1.32
EBV-miR-BART5-3p mimic group	3	12.26 ± 1.63^∗^
EBV-miR-BART5-3p inhibitor group	3	67.85 ± 3.59^∗^
*F*		457.599
*P*		<0.001

Note: In contrast to the control group, ^∗^*P* < 0.05.

**Table 4:** Comparison of the number of invasive cells in each group $\left(\overset{¯}{\chi }\pm s\right)$.

Grouping	*n*	Number of invasive cells (pcs)
Control group	3	55.33 ± 6.11
EBV-miR-BART5-3p NC group	3	55.33 ± 6.51
EBV-miR-BART5-3p mimic group	3	69.00 ± 2.65^∗^
EBV-miR-BART5-3p inhibitor group	3	30.33 ± 3.79^∗^
*F*		30.878
*P*		<0.001

Note: Compared to the control subjects, ^∗^*P* < 0.05.

**Table 5:** Comparison of cell migration rates in each group $\left(\overset{¯}{\chi }\pm s\right)$.

Grouping	*n*	Mobility (%)
Control group	3	77.65 ± 8.52
EBV-miR-BART5-3p NC group	3	76.88 ± 8.03
EBV-miR-BART5-3p mimic group	3	89.53 ± 6.74
EBV-miR-BART5-3p inhibitor group	3	32.67 ± 5.96^∗^
*F*		34.482
*P*		<0.001

Note: ^∗^*P* 0.05 vs. the control group.

**Table 6:** Comparison of apoptosis proteins in each group $\left(\overset{¯}{\chi }\pm s\right)$.

Grouping	*n*	Bcl-2	Bax	Caspase-3
Control group	3	1.55 ± 0.27	0.54 ± 0.03	0.58 ± 0.06
EBV-miR-BART5-3p NC group	3	1.52 ± 0.28	0.55 ± 0.08	0.58 ± 0.08
EBV-miR-BART5-3p mimic group	3	1.67 ± 0.24	0.28 ± 0.06^∗^	0.29 ± 0.07^∗^
EBV-miR-BART5-3p inhibitor group	3	0.34 ± 0.06^∗^	1.58 ± 0.23^∗^	1.64 ± 0.24^∗^
*F*		23.377	61.282	58.928
*P*		<0.001	<0.001	<0.001

Note: Compared with the control group, ^∗^*P* < 0.05.

**Table 7:** Comparison of cell invasion and apoptosis proteins in each group $\left(\overset{¯}{\chi }\pm s\right)$.

Grouping	*n*	MMP-2	MMP- 9
Control group	3	1.75 ± 0.23	1.69 ± 0.27
EBV-miR-BART5-3p NC group	3	1.77 ± 0.28	1.68 ± 0.29
EBV-miR-BART5-3p mimic group	3	1.96 ± 0.31	1.98 ± 0.28
EBV-miR-BART5-3p inhibitor group	3	0.51 ± 0.11^∗^	0.49 ± 0.10^∗^
*F*		22.352	21.178
*P*		<0.001	<0.001

Note: Compared with the control group, ^∗^*P* < 0.05.

## Data Availability

The data used to support the findings of this study are included within the article and in the supplementary data file.
